# Arabinogalactan from *Anacardium occidentale* Exudate as a Natural Emulsifier: A Sustainable Approach to Nanoemulsion Stabilization

**DOI:** 10.1021/acsomega.6c01433

**Published:** 2026-04-30

**Authors:** Elisandra Cibely Cabral de Melo, Alana Rayanne Araújo Freitas, Antonio Carlos da Silva Junior, Maria Isabela Ferreira de Araújo, Wilson Barros Júnior, Daniela Maria do Amaral Ferraz Navarro, Marthyna Pessoa de Souza, Kátia Alves Ribeiro, Maria das Graças Carneiro- da-Cunha, Paulo Antônio Galindo Soares

**Affiliations:** † Department of Biochemistry, 28116 Federal University of Pernambuco, Professor Moraes Rêgo Avenue, s/n, Cidade Universitária, Recife, Pernambuco 50670-420, Brazil; ‡ Department of Physics, Federal University of Pernambuco, Professor Luiz Freire Avenue, s/n, Cidade Universitária, Recife, Pernambuco 50670-901, Brazil; § Department of Fundamental Chemistry, 28116 Federal University of Pernambuco, Professor Luiz Freire Avenue, s/n, Cidade Universitária, Recife, Pernambuco 50670-901, Brazil; ∥ Keizo Asami Institute-iLIKA, Federal University of Pernambuco, Professor Moraes Rêgo Avenue, 1235, Cidade Universitária, Recife, Pernambuco 50670-900, Brazil

## Abstract

Oil/water nanoemulsions are effective systems for delivering hydrophobic compounds in aqueous media, provided that stable formulations are achieved. In this study, arabinogalactan extracted from *Anacardium occidentale* exudate was evaluated as a natural emulsifier for the production of nanoemulsions. Its performance was compared with that of guar gum, a widely used polysaccharide emulsifier. Nanoemulsions were prepared using spontaneous emulsification (magnetic stirring) and mechanical ultrahomogenization (Ultra-Turrax). The systems were characterized in terms of droplet size (DS), polydispersity index (PDI), zeta potential (ζ-potential), rheological behavior, and morphology by Transmission Electron Microscopy (TEM). Formulations produced by spontaneous emulsification with arabinogalactan (Ae4) showed droplet sizes below 40 nm, while guar gum systems (GGe4) presented sizes below 100 nm. Both systems exhibited PDI values lower than 0.5, indicating moderate to good homogeneity. A strong negative correlation (r < −0.8) was observed between polysaccharide concentration and droplet size, while a moderate correlation was found with ζ-potential, whose values close to neutrality (∼0 mV) suggest that steric stabilization is the dominant mechanism in arabinogalactan-stabilized systems. TEM analysis confirmed the nanoscale droplet size and revealed a more uniform distribution of Ae4 compared to GGe4, indicating improved stability. Notably, nanoemulsions stabilized with arabinogalactan remained stable for up to 70 days, with reduced droplet agglomeration over time compared to those stabilized with guar gum. These findings highlight the novelty of using arabinogalactan from *A. occidentale* as an efficient natural emulsifier. At higher concentrations and under spontaneous emulsification, it produces smaller droplets (up to ∼60% reduction) and enhances long-term stability. This polysaccharide represents a promising, safe, and sustainable alternative for applications in the food, cosmetic, and pharmaceutical industries.

## Graphical abstract

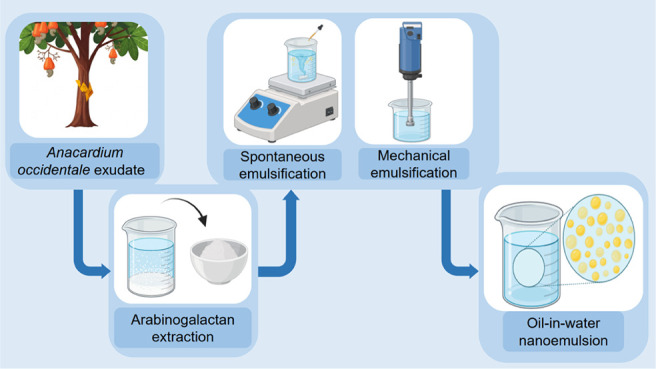

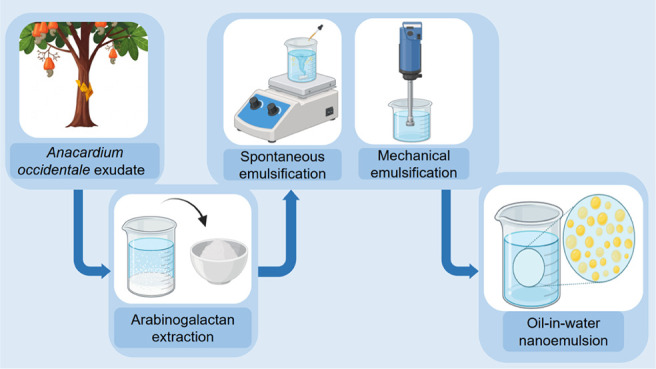

## 1 Introduction

The search for new, abundant, and sustainable natural emulsifiers has become strategically important in the bioeconomy, particularly for the development of food products.^1^ Emulsifiers are additives added to biphasic foods—that is, foods composed of an oil phase and an aqueous phase—such as sauces, creams, beverages, and bakery products, to maintain the stability of the dispersed phase, preventing its aggregation and phase separation.^2^ This type of food is called an emulsion, and in it, the dispersed phase is present as droplets in the continuous phase. This arrangement increases the interfacial area of the system, which consequently increases the free energy of the system, making emulsions thermodynamically unstable by nature.

In this context, emulsifiers play an important role in maintaining the stability of the dispersed phase within the continuous phase over time through steric hindrance, electrostatic repulsion, and/or alteration of the continuous-phase viscosity.^3^ The physicochemical characterization of emulsions, particularly the measurement of droplet size, polydispersity index (PDI), and ζ-potential, is essential for predicting stability and functionality, elucidating the dominant stabilization mechanisms, and identifying possible technological applications.

Among the most widely used natural emulsifiers, guar gum, extracted from the seeds of *Cyamopsis tetragonolobus*,^4^ stands out for its effectiveness and wide global application. However, its production is concentrated in specific Asian regions, such as India, which accounts for approximately 80% of global commercial guar gum.^5^ This geographic concentration imposes constraints on supply stability, pricing, and sustainability, highlighting the need for alternative sources.

In parallel, the global emulsifier market has experienced steady growth, driven by the increasing demand for processed products that utilize natural ingredients (green labels). However, Brazil imports more of these products than it exports.^6^ Therefore, the discovery of new emulsifier molecules is an interesting alternative to meet demand in the domestic market, which is expected to generate approximately 4.5 billion dollars in emulsifier purchases per year by 2028.^7^ This imbalance underscores a critical opportunity to explore native resources as competitive and sustainable alternatives.

In this sense, Brazil, renowned for its biodiversity and abundant plant resources, can be a source of valuable raw materials to diversify the emulsifiers available on the market, particularly from common and economically exploited plants in the country, such as the cashew tree (*Anacardium occidentale*). The cashew tree, widely cultivated in Brazil for the production of nuts and the pseudofruit known as cashew,^8^ produces an exudate originating from the healing process after a physical stress, which is rich in an arabinogalactan.^9^ Arabinogalactans are nontoxic, branched polysaccharides that have been demonstrated to have emulsifying activity in studies using diverse sources, such as corn^10^ and peaches.^11^ Additionally, it is the main component of another commercial emulsifier, the arabic gum.^12^

The study of the emulsifying potential of cashew arabinogalactan represents a significant step forward in the valorization of local resources, aligning with principles such as the bioeconomy and the sustainable exploitation of biodiversity for economic and social benefits. Furthermore, developing a local product can reduce dependence on imported raw materials, thereby increasing the resilience of the local production chain and fostering regional development, as well as the rational use of Brazil’s natural resources. To contribute more solidly to the bioeconomy approach, it is necessary to develop viable techniques for implementing the local industry. Therefore, in addition to analyzing the type of natural polysaccharide to replace imported ones, it is also necessary to study methods that produce more stable emulsions and can be more easily disseminated.

Additionally, most industrial emulsification processes rely on high-energy methods, such as mechanical ultrahomogenization, which, although effective, are energy-intensive and may limit scalability under sustainable production frameworks.^13^ In contrast, low-energy approaches, such as spontaneous emulsification, offer a promising alternative for producing stable nanoemulsions with reduced energy input. However, the effectiveness of natural polysaccharides under such conditions remains underexplored. To address these gaps, this study evaluates the emulsifying potential of purified arabinogalactan from *Anacardium occidentale* exudate within the broader context of natural emulsifiers. Guar gum was selected as a natural benchmark due to its established industrial use, while Tween 80 was included as a synthetic reference owing to its high emulsification efficiency and frequent application in nanoemulsion systems.

In summary, exploring new Brazilian sources of emulsifiers, such as arabinogalactan from cashew gum, alongside simplified processing techniques, supports the advancement of a sustainable and competitive bioeconomy. This study aims to determine the optimal conditions for using arabinogalactan from *Anacardium occidentale* in oil-in-water emulsions and to demonstrate its chemical similarity to commercial gums. For the first time, the emulsifying potential of purified arabinogalactan from *A. occidentale* exudate is evaluated, extending previous studies limited to the crude material. The purified exudate is proposed to exhibit enhanced emulsifying performance due to its higher polysaccharide content.

## 2 Materials and Methods

### 2.1 Materials

The gum exuded from the *A. occidentale* L. (cashew) tree was collected from the southern coast of Pernambuco State, Northeastern Brazil. Guar gum was purchased from Dinâmica Química Contemporânea LTDA, Brazil, and used without prior purification. Tween 80 (polysorbate 80, analytical grade) was purchased from Neon, São Paulo, Brazil, and glycerol (analytical grade) from Vetec, Rio de Janeiro, Brazil. Soybean oil (commercial food-grade, Mazola brand) was purchased from a local market (Recife, Pernambuco, Brazil). All other reagents used were of analytical grade and used as received.

### 2.2 Extraction of Arabinogalactan from *Anacardium occidentale* L. Gum

Arabinogalactan extraction was performed according to the method described by Souza et al.^14^ Briefly, the raw gum (in its natural state) was manually cleaned and ground. Samples of 20 g were dissolved in distilled water (100 mL) under magnetic stirring (200 rpm) for 2 h at room temperature (25.0 ± 0.1 °C). The obtained solution was filtered through a voile fabric, then filtered again using a screen-printing fabric (90 threads), and precipitated with ethanol (1:3 v/v). The precipitate was dissolved in distilled water, filtered through screen-printing fabric (110 threads), and finally dried at 30 °C, named arabinogalactan, and stored at room temperature (25.0 ± 0.1 °C). The selected extraction conditions were based on previously optimized protocols to ensure efficient polysaccharide recovery while preserving structural integrity. The arabinogalactan and commercial guar gum were subjected to total carbohydrate and protein content determination using the methods described by Dubois et al.^15^ and Bradford et al.,^16^ and galactose and bovine serum albumin standard curves (490 and 595 nm, respectively).

#### 2.2.1 Size Exclusion Chromatography

The purity and molar mass distribution of the arabinogalactan extracted were analyzed by gel permeation chromatography (GPC) using a Shimadzu LC-2050C system equipped with a refractive index detector (RID-10A). The analysis was conducted on a Polysep Linear column (300 × 7.8 mm) using a 0.1 mol·L^–1^ aqueous NaNO_3_ solution as eluent. Experimental conditions included a temperature of 30 °C, a flow rate of 1.0 mL·min^–1^, and an injection of 50 μL of the sample. The system calibration was performed using pullulan standards with different molecular weights (5 kDa, 12 kDa, 25 kDa, 50 kDa, and 80 kDa). A third-order polynomial fitted to the concentration results (logM versus retention time) was used to estimate the molecular weight of the sample, which was calculated using the equation below:

LogM=14.33638 −
1.12336×Ve

Being Ve, the elution volume.

### 2.3 Preparation of Oil-In-Water (O/W) Emulsions

Emulsions containing arabinogalactan and *guar gum* at concentrations of 2 and 4 mg/mL were produced. The emulsions were prepared using two distinct methodologies: the spontaneous emulsification method,^17^ with modifications, and the mechanical ultrahomogenization method employing an Ultra-Turrax (T-10, IKA, Germany). The polysaccharide concentrations were selected based on literature reports and preliminary optimization to ensure the formation of stable nanoemulsions while allowing the evaluation of polysaccharide contribution. The stability of the emulsions was evaluated over 70 days by analyzing the droplet size, polydispersity index (PDI), and ζ-potential on days 1, 7, 28, 49, and 70. The emulsions that presented droplets with a smaller average size and a lower polydispersity index (PDI) were subjected to rheological characterization on days 1 and 70 and evaluation by transmission electron microscopy on day 70. Samples from both methods were produced in triplicate, and the tests were conducted in triplicate for each repetition. The average ± standard deviation of the results obtained in each repetition was considered in the statistical analysis.

#### 2.3.1 Spontaneous Emulsification

The emulsions were prepared using Tween 80 (11.25% v/v) and glycerol (3.75% v/v) as adjuvants, whose concentrations were chosen according to the method described byJafarizadeh-Malmiri Jafarizadeh-Malmiri et al.^17^ with modifications, to optimize the application of the polysaccharides. Initially, both Tween 80 and glycerol were mixed by magnetic stirring (752, Fisatom, Brazil) at 500 rpm for 5 min, then soybean oil (1% v/v) was added. The mixture was stirred again for 15 min. Finally, the aqueous phase (84% v/v) was added, consisting of Milli-Q water with the addition of arabinogalactan (A) or guar gum (GG) at concentrations of 2 or 4 mg/mL. The mixture was stirred for 30 min. The samples were named as Ae2, Ae4, GGe2, and GGe4. Additionally, a control emulsion without polysaccharides was produced to evaluate the isolated emulsifying activity of Tween 80 and glycerol, named Ct.

#### 2.3.2 Mechanical Ultrahomogenization

To prepare the emulsions, soybean oil and a polysaccharide solution at concentrations of 2 or 4 mg/mL were combined in a 1:100 (v/v) ratio and homogenized individually using an Ultra-Turrax (T-10, IKA, Germany) at 20,000 rpm for 5 min. The samples were named Am2, Am4, GGm2, and GGm4. Tween 80 and glycerol were not included in this method due to preliminary observations of excessive foam formation, which interfered with analytical measurements. Additionally, the production of a control group containing only water and oil was not prepared due to the inherent instability of the system, as its phases separated immediately after the mechanical stirrer was turned off.

### 2.4 Emulsions Characterization

#### 2.4.1 Dynamic Light Scattering and ζ-Potential

The size of the droplets suspended in the emulsion, their respective polydispersity index (PDI), and ζ-potential were measured using a Zetasizer Nano ZS90 (ZEN3690, Malvern Instruments, U.K.). For this purpose, 1 mL of the emulsion was deposited in a glass cuvette (PCS1115). After verifying the droplet size (nm) and PDI using the dynamic light scattering,^18^ a universal “Dip” adapter (ZEN1002) was connected to the cuvette to measure the ζ-potential. The values were calculated using the Smoluchowski equation^19^ and the refractive index of soybean oil (1.47) at 20 °C with a laser at a fixed angle of 90°.^20^ The results were expressed as mean ± standard deviation of three measurements.

#### 2.4.2 Rheological Analysis

The rheological properties of Ae4, GGe4, and Ct emulsions were measured in a stress-controlled rheometer (Anton Paar MCR 102, Austria) equipped with a cone and plate cell (50 mm diameter with 1° and 101 μm truncation gap). Analyses were performed at room temperature (25.0 ± 0.1 °C). Flow curves were obtained by measuring sample stress response as a function of shear rate, ranging from 10 to 600 s^–1^. Data were depicted as apparent viscosity as a function of shear rate. Thirty data points were acquired per run.

#### 2.4.3 Transmission Electron Microscopy

The microscopic appearance of the Ae4 and GGe4 emulsions was analyzed using a Morgagni 268D microscope (FEI, USA) in brightfield mode and an accelerating voltage of 80 keV.

### 2.5 Statistical Analysis

Data were obtained in triplicate and evaluated by ANOVA followed by Tukey’s test (*p* ≤ 0.001), and by correlation analysis of polysaccharide concentration (mg/mL) and droplet size (nm), PDI, and ζ-potential (mV) of the emulsions on days 1 and 70. GraphPad Prism 8.0 (GraphPad Software, Inc.) was used for this purpose.

## 3 Results and Discussion

### 3.1 Purity of the Arabinogalactan

Considering the chemical structure, the polysaccharide from *A. occidentale* exudate shows variation in monosaccharide proportions depending on the extraction site; however, its chemical composition does not vary significantly.^21^ The *A. occidentale* gum found in Brazil is composed of a central galactose chain (73%) and terminal units of glucose (11%), arabinose (5%), rhamnose (4%), and glucuronic acid (6.3%).^22^

The *A. occidentale* arabinogalactan had a total carbohydrate content of 75.0 ± 0.8% (w/w) and a protein content of 4.38 ± 0.1% (w/w), whereas guar gum had a total carbohydrate content of 91.06 ± 0.2% (w/w) and a protein content of 5.27 ± 0.1% (w/w). Guar gum is mainly composed of galactomannans. In the literature, its composition is described as having a total carbohydrate content between 83.3 and 87.5% and a protein content between 3.5 and 5.0%, and its use as a stabilizer in o/w emulsions is due to the large amount of polysaccharides that promote stabilization by altering the viscosity of the aqueous phase.^23^ And despite the small amount of protein in guar gum, its presence is important to ensure the anchoring of the polysaccharide portions at the oil–water interface, which increases the stability of the system and confers emulsifying activity to this compound.^24^ Therefore, the chemical structure similarity between guar gum and arabinogalactan, in terms of the polysaccharide:protein ratio, suggests the possible technological function of arabinogalactan as a stabilizing or emulsifying agent.

The arabinogalactan extracted from *A. occidentale* showed a higher amount of polysaccharides and a lower amount of proteins compared to the arabinogalactan extracted from *Basella albaBasella alba*, whose polysaccharide portion represented 53.07 ± 5.01% of the sample and was mainly composed of arabinose, galactose, and glucuronic acid, while the protein portion represented 41.53 ± 2.48%.^25^ Despite the differences in the proportion between polysaccharides and proteins, these results reveal the chemical similarity between the samples, composed of similar monosaccharides, although they come from different sources. Similarly to what was observed in this study, the total content of water-soluble carbohydrates in *Urtica canabina* was up to 73%, with rhamnose, arabinose, glucose, and galactose among the identified monomers.^26^

The purity of the arabinogalactan extracted from *A. occidentale* was better explained by size-exclusion chromatography (Figure 1), which indicated that the material extracted from the exudate of *A. occidentale* is composed of 90.91% of a fraction with a molecular weight of 4.12 × 10^2^ Da and smaller fractions. These results corroborate the total carbohydrate and protein dosage tests performed, as they reinforce the existence of a more concentrated fraction with high molecular mass.

**Figure 1:**
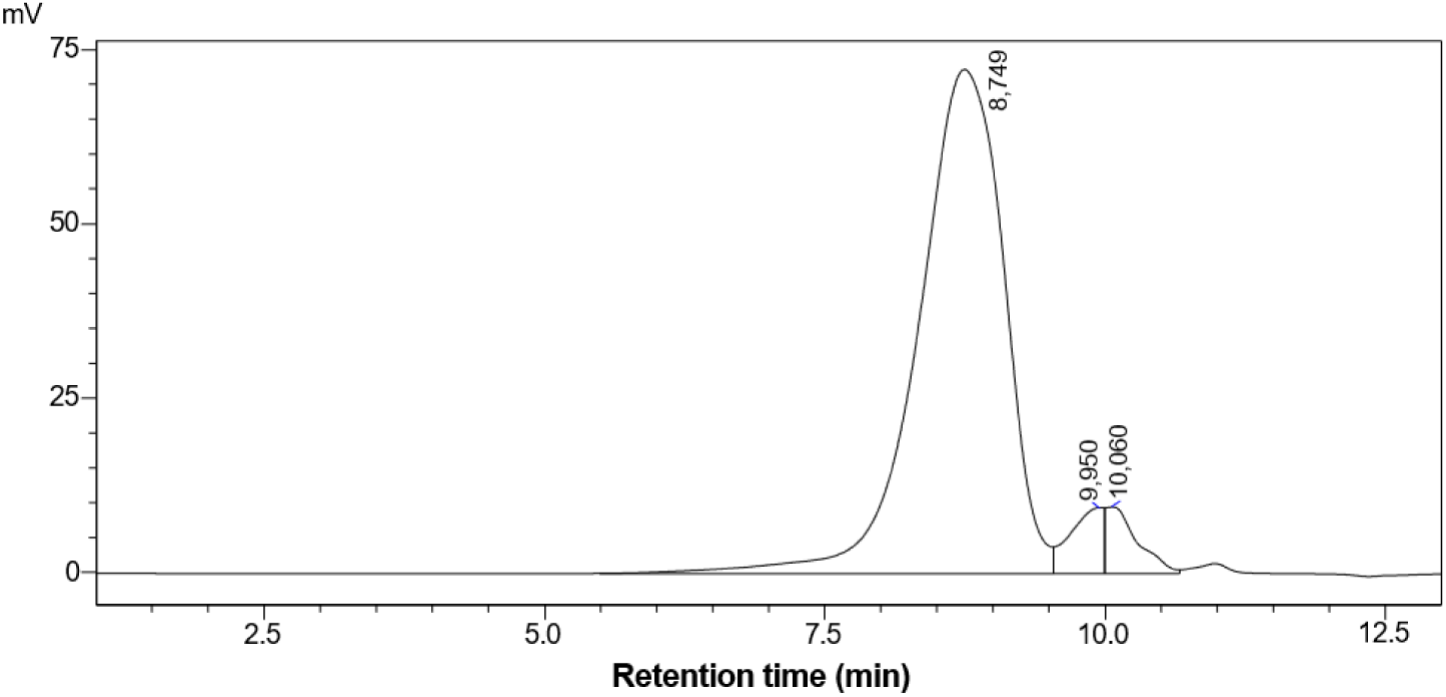
Size-exclusion chromatography profile of the arabinogalactan extracted from *Anacardium occidentale*.

Arabinogalactans can have high molecular weight, such as that extracted from *Codonopsis pilosula*, whose molecular weight of 1.29 × 10^7^ Da was higher than that obtained in this study.^27^ The arabinogalactan extracted from wood byproducts of *Larix sibirica* Ledeb also had a higher molecular weight than that observed in this study, with 19.805 Da.^28^

### 3.2 Impact of Spontaneous Emulsification and Mechanical Ultrahomogenization Processes on the Shelf Life of Emulsions

This study evaluated the emulsifying potential of arabinogalactan extracted from *A. occidentale* exudate compared to commercial guar gum, a galactomannan polysaccharide generally extracted from the endosperm of *Cyamopsis tetragonolobus* ^29^ and commercially used as an emulsifier to prevent phase separation in foods and cosmetics.^30^ The stability of the emulsions was assessed through their droplet size, PDI, and ζ-potential.

Promising results were observed for emulsion Ae4, whose droplet size (<20 nm) was smaller compared to emulsions Ct (<2000 nm) and Ae2 (<1000 nm). This stability was maintained over the course of 70 days (Table 1).

**Table 1:** Droplet Size (nm) of Emulsions Produced with Arabinogalactan Extracted from the Exudate of *A. occidentale* (A), Guar Gum (GG) as a Positive Control, and Water with Tween 80 (Ct) by Spontaneous Emulsification (E) and by Mechanical Ultrahomogenization (M)^a^

Droplet Size (nm)					
Day	1	7	28	49	70
Ae2	1037.3 ± 6.4^a^	973.2 ± 25.1^a^	174.5 ± 17.7^b^	241.2 ± 128^b^	334.5 ± 102.1^b^
Ae4	31.7 ± 12.1^a^*	15.8 ± 1.4^a^*	12.5 ± 0.5^a^*	12.5 ± 0.3^a^*	13.2 ± 0.4^a^*
GGe2	1030.2 ± 334.4^a^	826.2 ± 30^a^	1117 ± 253.1^a^	499.7 ± 1.3^b^	1035.5 ± 66.5^a^
GGe4	21.3 ± 1.3^a^*	22.1 ± 5^a^*	21.2 ± 3.1^a^*	21.6 ± 5.4^a^*	78.3 ± 6.3^a^*
Ct	1405 ± 182.9^a^	1028.9 ± 410.9^a^	1159.1 ± 203.2^a^	1969 ± 302^a^	561 ± 107.3^b^
Am2	705.6 ± 33.6^a^	857.3 ± 46.3^a^	1087.7 ± 89.4^a^	843 ± 250^a^	585.6 ± 28.1^a^
Am4	285.8 ± 10.6^a^	1138 ± 35.7^b^	968.8 ± 15.6^b^	994.8 ± 6.7^b^	1117 ± 119.7^b^
GGm2	4222 ± 126^a^	3845.7 ± 556.3^a^	2622.7 ± 236^b^	2616 ± 122.1^b^	2359.7 ± 35.1^b^
GGm4	1061 ± 42.6^a^	2461 ± 54.3^b^	3966 ± 92.8^c^	3898 ± 189.8^c^	3994.3 ± 24.7^c^

a Different superscript lowercase letters indicate statistical difference (*p* < 0.001) between days, and *­(*p* < 0.001) indicates statistical difference between samples on the same day.

The GGe4 emulsion produced with guar gum under the same conditions as Ae4 showed similar results to those of arabinogalactan; however, there was a significant increase on day 49. The samples Ae4 and GGe4 presented sizes below 20 and 100 nm, respectively, on day 70, while the formulations with lower concentrations, Ae2 and GGe2, presented sizes between 300 and 1000 nm (Table 1). This suggests that the reduction in polysaccharide concentration led to an increase in droplet size in samples produced by spontaneous emulsification. This behavior can be mechanistically explained by the increased availability of polysaccharide molecules at higher concentrations, which enhances interfacial adsorption during emulsification. A greater surface coverage reduces interfacial tension and enables the rapid stabilization of newly formed droplets, preventing recoalescence. In addition, the formation of a thicker steric barrier around the droplets limits their aggregation over time. At lower concentrations, insufficient interfacial coverage leads to partial stabilization, favoring droplet coalescence and resulting in larger average droplet sizes.

The correlation between polysaccharide concentration and emulsion quality was also evaluated in this work, and the results are presented in Table 2. This result was based on Pearson’s coefficient, whose values between |1.0| and |0.5| represent a strong correlation, between |0.5| and |0.3|, a moderate correlation, between |0.3| and |0.1|, a weak correlation, and below |0.1|, no correlation.^31^

**Table 2:** Correlation between Polysaccharide Concentration and Droplet Size, Polydispersity Index (PDI), and ζ-Potential of Emulsions Prepared with Arabinogalactan from *Anacardium occidentale* Exudate and Guar Gum Expressed by Pearson’s Coefficient (R)

Day	Droplet Size	PDI	ζ-Potential
1	–0.55	–0.17	–0.34
70	0.07	–0.24	0.04

According to Table 2, the concentration of the tested polysaccharides had a strong influence on the reduction in droplet size on day 1 (r = −0.55). Obtaining smaller droplets increases emulsion stability, as smaller, more uniformly sized droplets hinder their coalescence and, consequently, phase separation.^32^

The samples produced by mechanical ultrahomogenization had larger droplet sizes than those obtained by spontaneous emulsification (Table 1). The emulsion produced by mechanical ultrahomogenization uses only agitation at approximately 20,000 rpm to form the emulsion structure. Conversely, spontaneous emulsification uses magnetic agitation (500 rpm) and is less vigorous. It is important to note that the comparison between spontaneous emulsification and mechanical ultrahomogenization involves differences not only in energy input but also in formulation composition, particularly the presence of surfactant adjuvants. Therefore, the observed differences should be interpreted as resulting from distinct formulation–process systems, rather than as a direct comparison of emulsification methods.

In addition to the differences between techniques, differences were observed between the samples produced by mechanical ultrahomogenization. Those produced with arabinogalactan had smaller droplet sizes (approximately 1000 nm, Table 1) compared to those produced with guar gum (between 2300 and 4000 nm, Table 1). These results indicate that arabinogalactan exhibited greater stabilizing activity than the commercial control, producing emulsions with smaller droplets.

The droplet size distribution is analyzed through PDI. In this work, the emulsions prepared with arabinogalactan (Ae4, Am2, and Am4) exhibited PDI values below 0.5 over 70 days (Table 3), with Ae4 standing out, showing a statistically significant difference compared to Ct and Ae2. In parallel, for the positive control, only GGe4 presented a PDI of 0.5 ± 0 (Table 3); this result indicates that the type of polysaccharide strongly influenced the stability of the system, since, using the same technique and surfactant concentration, it was not possible to obtain an emulsion as stable with guar gum as those obtained with arabinogalactan.

**Table 3:** Polidispersity Index (PDI) of Emulsions Produced with Arabinogalactan Extracted from the Exudate of *A. occidentale* (A), Guar Gum (GG) as a Positive Control, and Water with Tween 80 (Ct) by Spontaneous Emulsification (E) and by Mechanical Ultrahomogenization (M)^a^

Polidispersity Index (PDI)					
Dia	1	7	28	49	70
Ae2	0.949 ± 0^a^	0.723 ± 0.1^b^	0.889 ± 0.2^a^	0.916 ± 0.1^a^	0.829 ± 0.2^a^
Ae4	0.352 ± 0^a^*	0.337 ± 0^a^*	0.368 ± 0^a^*	0.362 ± 0^a^*	0.367 ± 0^a^*
GGe2	0.247 ± 0.1^a^*	0.236 ± 0.1^b^*	0.953 ± 0^a^	0.982 ± 0^a^	0.788 ± 0.2^a^
GGe4	0.440 ± 0^a^*	0.374 ± 0^a^*	0.360 ± 0^b^*	0.372 ± 0^b^*	0.527 ± 0^b^*
Ct	0.688 ± 0.1^a^	1.000 ± 0^bC^	1.000 ± 0^b^	1.000 ± 0^b^	1.000 ± 0^b^
Am2	0.231 ± 0^a^*	0.293 ± 0^b^*	0.371 ± 0^b^*	0.308 ± 0.1^b^*	0.295 ± 0^b^*
Am4	0.371 ± 0^a^*	0.239 ± 0^a^*	0.511 ± 0^a^*	0.569 ± 0^b^*	0.484 ± 0.1^b^
GGm2	0.506 ± 0.1^a^*	0.462 ± 0^a^*	0.573 ± 0^a^*	1.000 ± 0^b^	1.000 ± 0^b^
GGm4	0.440 ± 0^a^*	1.000 ± 0^b^	1.000 ± 0^b^	1.000 ± 0^b^	1.000 ± 0^b^

a Different superscript lowercase letters indicate statistical difference (*p* < 0.001) between days, and *­(*p* < 0.001) indicates statistical difference between samples on the same day.

The polydispersity index indicates the dispersion of the emulsion’s droplet sizes. The closer to 1.0, the greater the number of droplets of different sizes, and the closer to 0.0, the smaller the variation in droplet sizes. In other words, the more uniform the droplet size, the more stable the emulsion. Contact between droplets of very different sizes promotes coalescence processes, in which smaller droplets are incorporated into larger ones, which facilitates phase separation.^32^ Therefore, obtaining emulsions with a PDI close to 0.0 is desirable for the development of stable emulsions.

Unlike the polysaccharide formulations, Ct presented a high PDI from day 1, with its PDI remaining at 1.0 throughout the remaining days (Table 3), indicating that emulsion stability depended primarily on the presence of polysaccharides and, second, on the type of polysaccharide used; in this case, arabinogalactan was the most prominent. The emulsification method did not directly influence the PDI, and emulsions with uniform droplet sizes were obtained using both techniques^33^.

In addition to PDI, the ζ-potential is a parameter that can be used to elucidate the stabilization mechanism by assessing the presence or absence of charges on the particles, and to compare the stability of charged samples.^34^ This occurs because the ζ-potential is an indicator of the electrical charge at the interface of the emulsion droplets.^35^ Emulsions stabilized by unmodified polysaccharides tend to be neutral or slightly charged due to the nature of these molecules, indicating that the emulsion stabilization mechanism occurs to a lesser extent by electrostatic repulsion, which would cause a high ζ-potential (positive or negative), and to a greater extent by steric hindrance caused by the polysaccharide molecules at the oil–water interface.^36^

In this study, ζ-potential values of the samples were close to 0 mV (Table 4). The emulsions stabilized with the arabinogalactan from *Anacardium occidentale* exhibited droplet sizes in the nanometric range and maintained low polydispersity over the 70-day storage period, indicating a high degree of kinetic stability. Notably, these systems remained stable despite ζ-potential values close to neutrality, suggesting that electrostatic repulsion is not the primary mechanism of stabilization.

**Table 4:** ζ-Potential (mV) of Emulsions Produced with Arabinogalactan Extracted from the Exudate of *A. occidentale* (A), Guar Gum (GG) as a Positive Control, and Water with Tween 80 (Ct) by Spontaneous Emulsification (E) and by Mechanical Ultrahomogenization (M)^a^

ζ-Potential (mV)					
Dia	1	7	28	49	70
Ae2	–0.3 ± 0^a^	–0.3 ± 0^a^	–0.4 ± 0.1^a^	–0.4 ± 0.1^a^	–0.5 ± 0.1^a^
Ae4	–0.8 ± 0.1^a^	–0.5 ± 0^a^	–0.4 ± 0.1^a^	–0.5 ± 0.1^a^	–0.5 ± 0.1^a^
GGe2	–0.3 ± 0^a^	–0.3 ± 0^a^	–0.2 ± 0^a^	–0.5 ± 0^a^	–0.4 ± 0^a^
GGe4	–0.7 ± 0^a^	–0.6 ± 0^a^	–1.1 ± 0.1^a^*	–1.0 ± 0.2^a^	–1.3 ± 0.1^a^
Ct	–0.4 ± 0^a^	–0.5 ± 0^a^	–0.4 ± 0^a^	–0.6 ± 0^a^	–0.5 ± 0.1^a^
Am2	–8.5 ± 0.2^a^*	–8.7 ± 1^a^*	–9.4 ± 0.2^b^*	–9.4 ± 0.2^b^*	–8.9 ± 0.1^a^*
Am4	–0.9 ± 0.3^a^	–7.2 ± 0^b^*	–7.5 ± 0.1^b^*	–7.2 ± 0.9^b^*	–7.7 ± 0.1^b^*
GGm2	–5.3 ± 0.3^a^*	–1.6 ± 0.1^b^	–0.7 ± 0^c^	–1.2 ± 0.2^b^	–1.6 ± 0.2^b^
GGm4	–8.3 ± 0.1^a^*	–1.2 ± 0^b^	–1.1 ± 0.1^b^	–1.3 ± 0.2^b^	–1.8 ± 0.2^b^

a Different superscript lowercase letters indicate statistical difference (*p* < 0.001) between days, and *­(*p* < 0.001) indicates statistical difference between samples on the same day.

The use of neutral polysaccharides instead of charged polysaccharides was also responsible for increasing the stability of oil–water emulsions over time.^37^ Therefore, it is not possible to associate emulsion instability with a ζ-potential close to zero without considering the components and the emulsion production method.

In this context, the ζ-potential values (Table 4) should be interpreted as indicative of the limited contribution of electrostatic interactions, rather than as a determinant of emulsion stability. Instead, the observed stability is more consistently explained by a steric hindrance mechanism, in which the adsorption of arabinogalactan chains at the oil–water interface forms a hydrated interfacial layer that prevents droplet coalescence.

Comparatively, although guar gum also demonstrated emulsifying capacity, the emulsions stabilized with arabinogalactan showed smaller droplet sizes and a more uniform distribution, particularly under spontaneous emulsification conditions. This suggests that arabinogalactan may form a more effective interfacial layer, potentially due to its molecular structure and branching pattern, which favor steric stabilization. This performance was also associated with the presence of proteins in the sample, which were responsible for keeping the arabinogalactan adsorbed at the interface. The polysaccharide portions projected into the aqueous phase, allowing the formation of a steric layer that prevented the agglomeration of droplets.^38^ Also, considering the carbohydrate and protein content of the arabinogalactan, it is suggested that its emulsifying mechanism is associated with steric hindrance, as indicated by the ζ-potential, using the protein components of the material extracted from the exudate as an anchor for the polysaccharide at the oil–water interface.

This combination of proteins and polysaccharides is also observed in guar gum, which was identified as a protein-conjugated galactomannan.^39^ These similarities suggest a similar emulsification mechanism and support the quality of *A. occidentale* arabinogalactan as an emulsifier. Alkaline extracts of saccharin from beet pulp were produced in order to obtain protein–polysaccharide conjugates that showed emulsifying activity in oil-in-water emulsions.^40^ Similarly, chitosan and protein conjugates extracted from soybeans demonstrated good emulsifying activity, with potential applications in the food industry;^41^ these results corroborate the findings of this study.

In similar studies, emulsions produced with acetylated raw cashew gum presented a particle size larger than that obtained in this study, of 312.06 ± 12.57 nm, PDI of 0.447 ± 0.04, and ζ-potential of −46.00 ± 1.75 mV. Acetylation was employed to enhance the hydrophobicity of this compound, thereby increasing its efficiency as a stabilizer at the oil/water interface.^42^ Similarly, investigation of the use of polysaccharides extracted from *Ulva fasciata* obtained emulsions with sizes between 2280 and 2870 nm and ζ-potential that varied between approximately −40 and −45 mV depending on the preparation conditions,^43^ values much higher than those obtained by Ae4 (<20 nm, Table 1) and GGe4 (<100 nm, Table 1), reinforcing that different factors must be analyzed to classify an emulsion as stable.

Thus, the results of this study corroborate the literature review, as emulsions produced by spontaneous emulsification remained stable, without phase separation (Figure 2) and with a nanometric size after 70 days, even with a ζ-potential close to zero mV. Meanwhile, Am4, prepared by mechanical ultrahomogenization, despite its higher ζ-potential (approximately −8 mV, Table 3), exhibited a large droplet size (1117 ± 119.7, Table 1), in addition to phase separation (Figure 2), which highlights its inherent instability.

**Figure 2:**
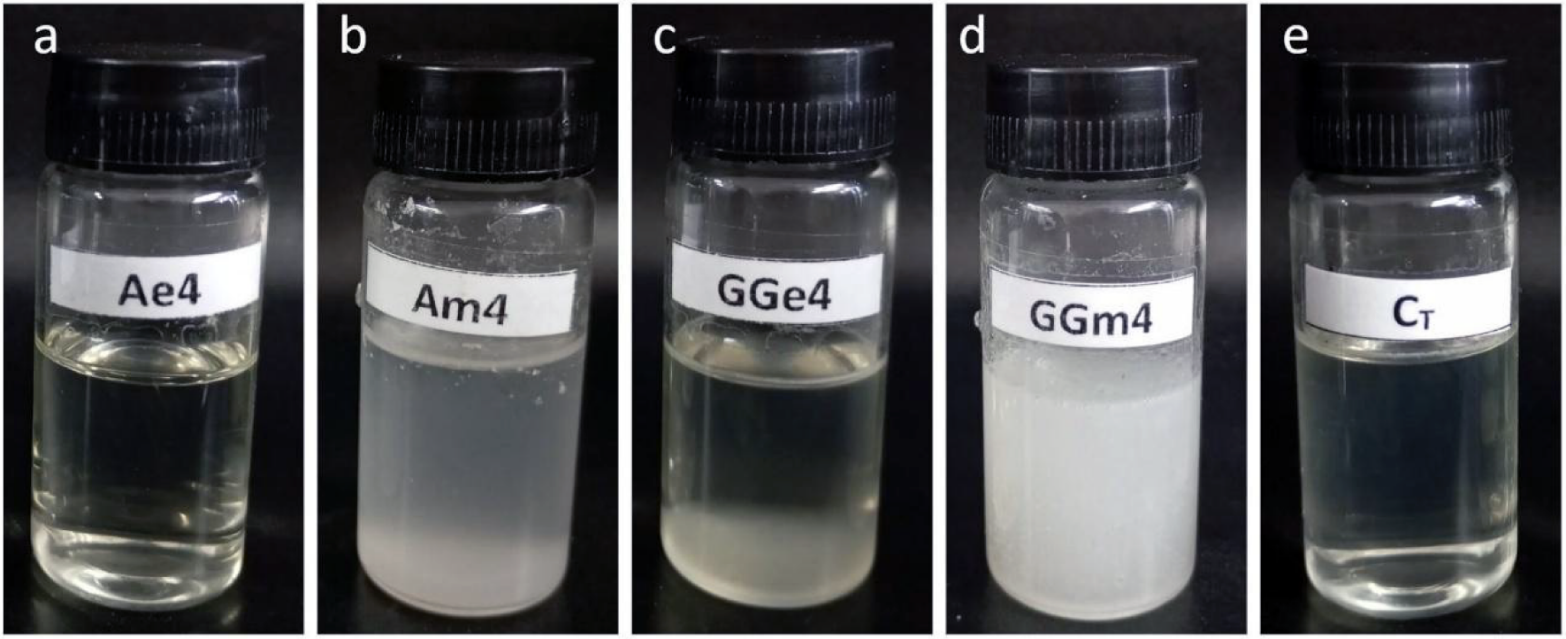
Macrometric aspect of emulsions produced with arabinogalactan from *A. Occidentale*by spontaneous emulsification (a) and mechanical ultrahomogenization (b), guar gum by spontaneous emulsification (c) and mechanical ultrahomogenization (d), and water with Tween 80 by spontaneous emulsification (e).

The ζ-potential was negatively influenced by the polysaccharide concentration on day 1 (r = −0.34), a correlation considered moderate.^31^ Thus, the increase in the polysaccharide concentration may be associated with a reduction in the negative value of the ζ-potential in the graph, that is, an increase in its absolute value. Therefore, to obtain a more stable emulsion, it is necessary to increase the polysaccharide concentration. The increase in the concentration of the copolymeric emulsifier, including *A. occidentale* gum, also increased the ζ- potential in emulsions, going from +17 to −39 mV.^44^ The polysaccharide extracted from the residues of *Apocynum venetum* L. produced emulsions with a ζ-potential around +40 mV,^45^ absolute values higher than those found in this work.

On day 70, the correlation between polysaccharide concentration and particle size and ζ-potential was positive and lower than on day 1, indicating that concentration did not significantly influence the other variables. PDI, however, remained inversely proportional, albeit at a higher level than on day 1, indicating that polysaccharide concentration had a greater influence on the results over time. A study by^42^ using acetylated raw cashew gum resulted in emulsions with a ζ-potential of −46.00 ± 1.75 mV. The authors concluded that the different degrees of acetylation and the ionic strength of the system influenced the results.

Emulsions stabilized by a copolymer formed by purified *A. occidentale* gum and poly-l-lactic acid were tested at different concentrations for droplet size, PDI, and ζ-potential. The authors observed that droplet size increased with increasing copolymer concentration, unlike the findings of this study.^44^ The study by Riana et al.^46^ indicated that increasing the concentration of *Meryta sinclairii* exudate reduced droplet size, a finding similar to that observed in this study. In this case, the authors explain that due to the bimodal size distribution, it is possible to suggest that the low gum concentration prevented the formation of a uniform interfacial coverage, leading to droplet recoalescence and an increase in their size.

In summary, it is important to note that the comparison between spontaneous emulsification and mechanical ultrahomogenization involves differences not only in energy input but also in formulation composition, particularly the presence of surfactant adjuvants. Therefore, the observed differences should be interpreted as resulting from distinct formulation–process systems, rather than as a direct comparison of emulsification methods.

### 3.3 Rheological and Microscopic Analysis of Selected Stable Nanoemulsions

Based on the results obtained, the formulations considered most stable, Ae4 and GGe4, as well as the negative control Ct, were selected to undergo evaluation of their rheological behavior and microscopic appearance. Among the samples analyzed (Figure 3), Ae4 exhibited greater stability in apparent viscosity over the 70 days compared to the positive control (GGe4), which showed the most significant variation in apparent viscosity.

**Figure 3:**
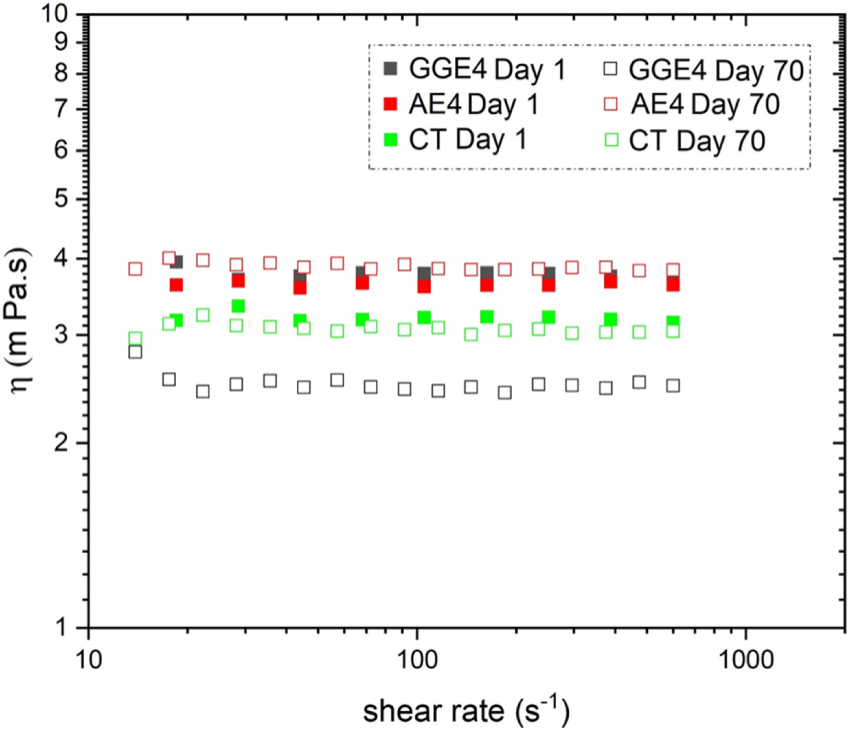
Apparent viscosity of emulsions containing arabinogalactan from *Anacardium occidentale* (A), guar gum (GG), and water added with Tween 80 (CT) on days 1 and 70.

The viscosity of the continuous phase directly influences the immobilization capacity of the dispersed phase droplets,^47^ especially in diluted emulsions, those with a dispersed phase concentration below 2%,^48^ such as those obtained in this work (0.5–1% v/v). Thus, the maintenance of apparent viscosity in the formulation containing arabinogalactan is a positive aspect, indicating the greater stability of this compound compared to commercial guar gum.

The emulsions obtained in this work exhibited Newtonian fluid behavior. Emulsions exhibiting this behavior can be prone to destabilization mechanisms such as gravity-induced separation, Ostwald ripening, and droplet coalescence.^49^ However, the dilute nature of the formulations hinders interaction between droplets and delays coalescence processes that would lead to phase separation. This dilute appearance is evident in Figure 4, where it can be observed that the droplets remain separated from each other, promoting relatively weak interactions.^50^

**Figure 4:**
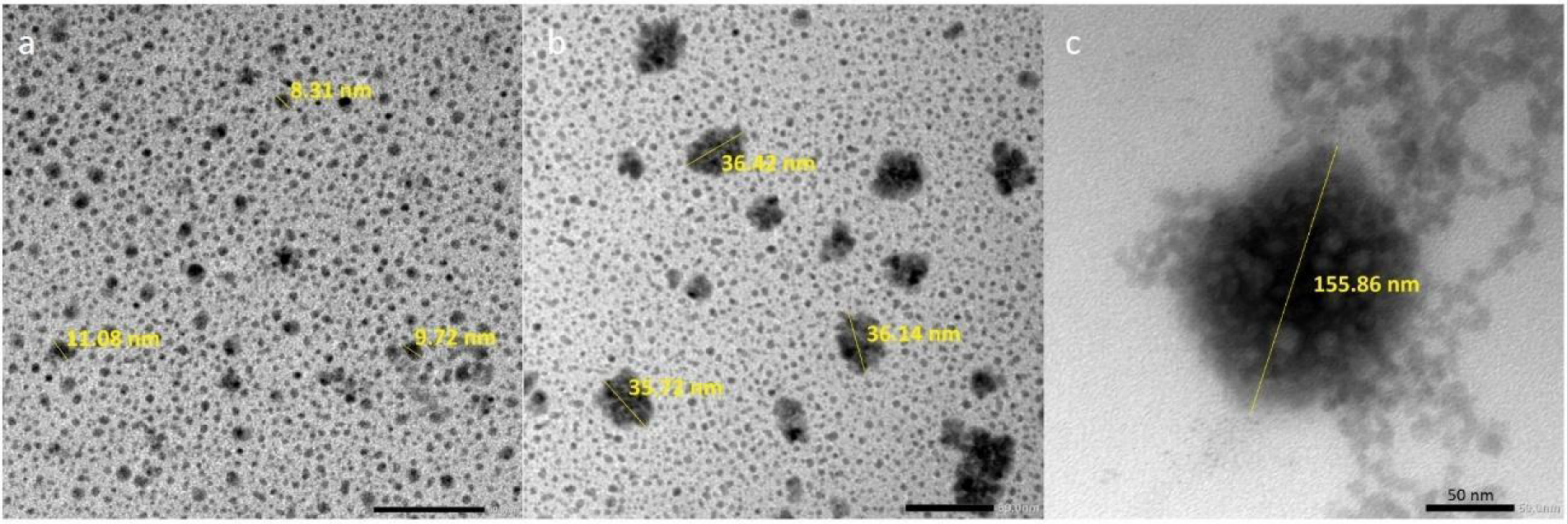
Transmission electron microscopy of emulsions produced by spontaneous emulsification and stabilized by (a) arabinogalactan from *A. occidentale* at 4 mg/mL (Ae4), (b) commercial guar gum at 4 mg/mL, and (c) negative control without stabilizer. The emulsions were prepared and analyzed 70 days after preparation.

In addition to the diluted appearance, microscopy confirmed the nanometric size of the Ae4 and GGe4 droplets, with 9.12 ± 0.48 and 22.24 ± 6.3 nm, respectively. The droplets identifiable in Ct presented sizes of 16.89 ± 1.31 nm, a smaller size than that obtained by DLS (approximately 1000 nm, Table 1). This discrepancy can be attributed to the agglomeration state of the droplets, as observed in Figure 4c, which may have been misidentified as a single droplet by the equipment. This agglomeration of droplets can also be observed to a lesser extent in the microscopy of GGe4 (Figure 4b), indicating the importance of a stabilizing component in the formulation. On the other hand, Ae4 presented a more uniform droplet distribution, corroborating the results obtained with PDI. Regarding this, the arabinogalactan from *Anacardium occidentale* produces significantly smaller droplet sizes (<20 nm) and maintains stability for up to 70 days, which compares favorably with several emerging polysaccharide-based emulsifiers reported in the literature. In addition, its near-neutral ζ-potential indicates a steric stabilization mechanism, which may offer advantages in systems where electrostatic interactions are limited (e.g., in high-ionic-strength media).

The emulsions that exhibited the most excellent stability were produced by spontaneous emulsification or self-emulsification. These terms describe techniques that use little or no mechanical energy to produce micro- and nanoemulsions.^51^ Figure 5 shows a representation of the emulsion preparation method by spontaneous emulsification and by mechanical homogenization, as well as the use of cosurfactants; the main difference between these techniques was the agitation speed.

**Figure 5:**
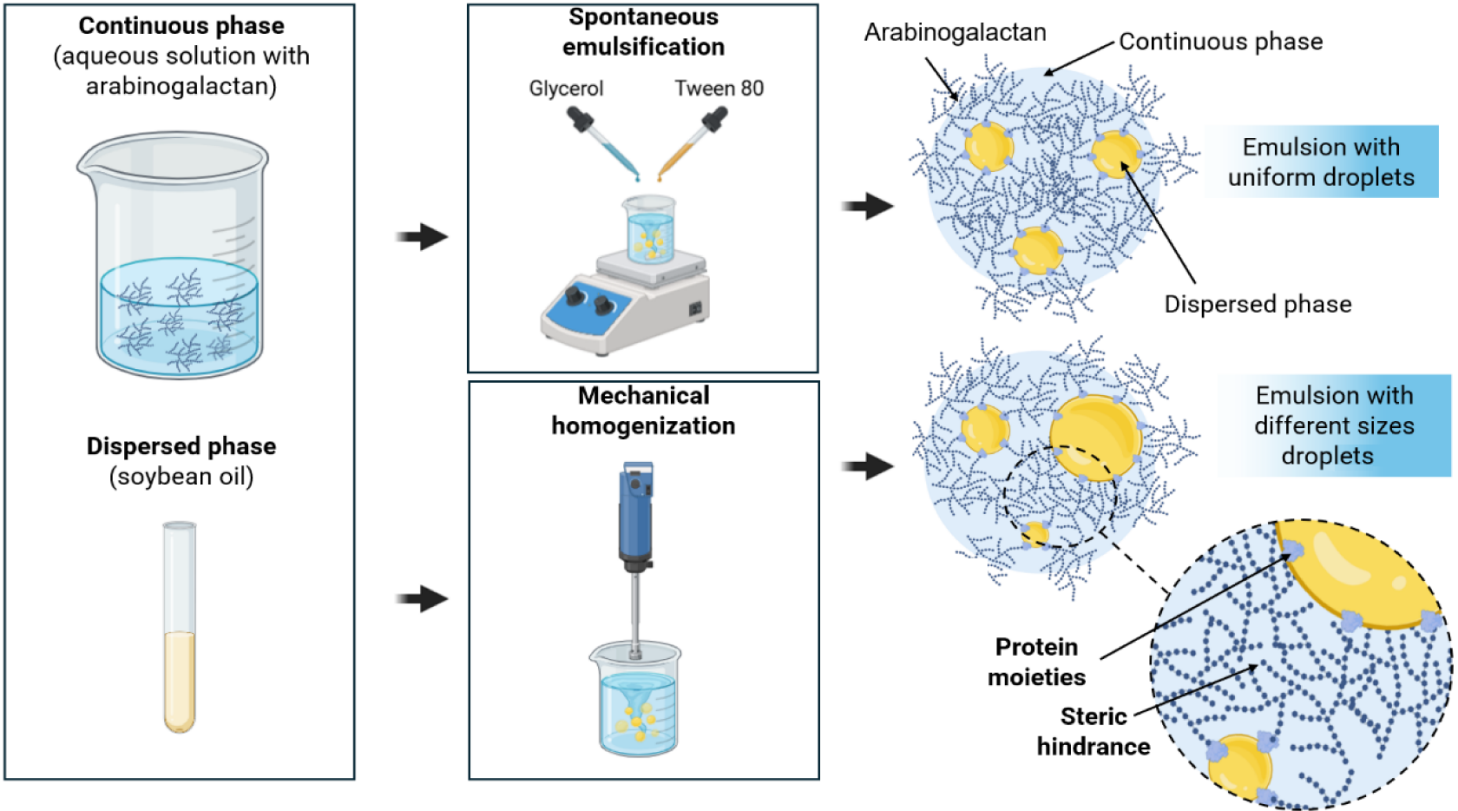
Graphical representation of the difference between spontaneous emulsification and mechanical homogenization techniques in the production of oil-in-water emulsions, and the steric hindrance emulsification mechanism promoted by the arabinogalactan.

At low stirring speed, spontaneous emulsification results in less loss of volatile materials, such as essential oils, and produces significantly smaller, more stable droplets compared to mechanical ultrahomogenization. In this regard, the use of a material with low intra- and intermolecular interactions, such as the polysaccharide from *A. occidentale* gum,^22^ ensures the integrity of the bioactive compound, allowing for a more reliable response. Additionally, the figure illustrates the adsorption of arabinogalactan at the interface, where protein moieties act as anchoring points to the oil phase, while the polysaccharide chains extend into the aqueous phase, forming a steric barrier that prevents droplet aggregation and coalescence. This representation supports the interpretation of the experimental results, particularly the near-neutral ζ-potential values and the observed long-term stability of the nanoemulsions.

This interpretation is supported by the strong negative correlation observed between polysaccharide concentration and droplet size, indicating that increasing polymer availability enhances interfacial coverage and reduces droplet coalescence. Furthermore, maintaining low PDI values over time reinforces the presence of an effective steric barrier that limits droplet aggregation even in the absence of significant electrostatic repulsion.

In addition to structural differences, emulsions prepared by mechanical ultrahomogenization are visually more turbid due to the larger droplet size; in this sense, the lack of Tween 80 influences the production of larger droplets.^52^ This characteristic is evident in Figure 2, which displays the final appearance of emulsions Ae4, Am4, GGe4, GGm4, and Ct.

On the other hand, emulsions prepared by spontaneous emulsification (Ae4, GGe4, and Ct) have a clearer appearance due to their smaller droplet size. The final appearance of the emulsion is a crucial factor in its commercial application, particularly considering the technological applications of emulsions that can be utilized in the food and cosmetics sectors. In this sense, Linke and Drusch^53^ analyzed the relationship between droplet size and turbidity of oil-in-water emulsions in beverages, observing that the larger the droplet size in the suspensions, the greater the turbidity. Considering recent trends toward obtaining clearer suspensions, one approach in this regard is to invest in techniques that produce finer and more stable emulsions, such as spontaneous emulsification.

The factors that influence spontaneous emulsification include the surfactant structure, initial concentration and location, the composition of the oil phase, the addition of a cosurfactant, such as Tween 80, and the use of a nonaqueous solvent, as well as salinity and temperature.^54^ In this work, the presence of Tween 80 alone was not sufficient for the emulsion to reach the nanometric scale and ensure stability, as can be observed in the Ct group, which presented droplet sizes greater than 500 nm and a PDI of 1.0 (Tables 1 and 3).

In the literature, Tween 80 and *Alpinia officinarum* pectin were used as emulsifiers in emulsions that presented droplet sizes of 303 ± 0.0 nm.^55^ Tween 80 and Span 80 were also tested for their emulsifying activity in multiple emulsions containing alginate and chitosan, the sizes obtained were equal to or greater than 337 ± 22 nm,^56^ results lower than those found in this work for the sample containing only Tween 80 and glycerol (>1000 nm, Table 1), but higher than those obtained by Ae4 (<40 nm, Table 1) and GGe4 (<100 nm, Table 1).

Tween 80 is a surfactant used in oil-in-water emulsion formulations^57^ commonly employed by the food and cosmetic industries.^58^ It produces emulsions with smaller droplet size and PDI compared to those made with Tween 20, 40, or 60.^59^ However, there are reservations regarding its use as an adjuvant for formulating products for this audience due to its toxicity.^60^ In contrast, *A. occidentale* gum is considered biocompatible and nontoxic^61,62^ and can be used as a strategy to reduce the concentration of Tween 80 during emulsion production.

Nanoemulsions are dispersions formed by immiscible and thermodynamically unstable liquids that require greater stabilization due to the higher free energy in the system.^63^ Some characteristics of nanoemulsions facilitate their use in food formulations, including their weak light scattering, promoting less visual interference in the product, greater thermodynamic stability, and reduced viscosity compared to conventional emulsions, preventing droplet aggregation and changes in food texture, in addition to increasing the bioavailability of lipophilic bioactive compounds.^64^

The use of nanoemulsions as carriers for hydrophobic compounds in hydrophilic media is a viable alternative; however, not all hydrophobic compounds can assume a stable structure in aqueous media. To solve this problem, it is possible to nanoemulsify a fixed oil containing a solubilized hydrophobic bioactive.^65,66^ The soybean oil used in this study, for example, is a source of essential fatty acids, including linoleic, palmitic, linolenic, and oleic acids.^67^ In food applications, these fatty acids, organized into nanometric micelles containing the hydrophobic bioactive, can be used to facilitate interaction with the enterocyte plasma membrane, allowing for greater diffusion of these bioactives into the cell.

The exudate of *A. occidentale* can be an alternative, from replacing commercial gums such as gum arabic or guar gum in the food and cosmetics industry, to developing drug delivery systems for the pharmaceutical industry. The reason is the similarity in the origin of these emulsifiers; both are derived from plants commonly found in arid regions, namely acacia, whose exudate yields arabic gum,^68^ and *C. tetragonoloba*, whose seeds are rich in polysaccharides, which are processed into guar gum.^69^ These materials differ from xanthan gum, for example, which is an exopolysaccharide extracted from the *Xanthomonas campestris* biomass,^70^ and therefore presents differences that make it difficult to compare with arabinogalactan. To verify the industrial application of arabinogalactan from *A. occidentale*, it is important to assess the feasibility of scaling up its production.

Regarding the raw material and in a local production context, *A. occidentale* is a plant widely distributed across South American countries,^71^ indicating ease of large-scale cultivation under local environmental conditions. The cashew tree produces exudate through incisions made in the stem, allowing specimens to be handled without replanting.

From an application perspective, the ability of arabinogalactan from *Anacardium occidentale* to produce stable nanoemulsions with small droplet sizes highlights its potential for use in food, cosmetic, and pharmaceutical systems, particularly for the encapsulation and delivery of hydrophobic bioactive compounds. However, the transition from laboratory to industrial scale may present challenges, including the need for cost-effective purification strategies, control of variability in raw material composition, and optimization of solvent usage during extraction. Addressing these factors will be essential to ensure the economic and technological feasibility of large-scale production.

Finally, given that the world production of gummy emulsifiers is still restricted to a few African and Asian countries, it is important to investigate new local sources of emulsifiers. Therefore, encouraging the study of local materials with superior potential to those currently on the market reduces external dependence on a material essential to the three most profitable industries in the world: food, pharmaceuticals, and cosmetics.

## 4 Conclusion

This study demonstrates, for the first time, the emulsifying potential of purified arabinogalactan from *Anacardium occidentale*, establishing it as a promising natural alternative to conventional emulsifiers. From a scientific perspective, the results show that arabinogalactan is capable of producing stable nanoemulsions with smaller droplet sizes (<20 nm) than those obtained with commercial guar gum, while maintaining stability for up to 70 days. In addition, the findings provide evidence that steric hindrance is the dominant stabilization mechanism, supported by near-neutral ζ-potential values and consistent droplet size distribution over time. From a practical standpoint, the combination of arabinogalactan at 4 mg/mL with spontaneous emulsification proved to be an efficient low-energy strategy for producing stable nanoscale emulsions. These results highlight its potential application in food, cosmetic, and pharmaceutical systems, particularly for the encapsulation and delivery of hydrophobic bioactive compounds. Furthermore, the use of a locally available and renewable raw material contributes to reducing dependence on imported emulsifiers and supports the development of a sustainable bioeconomy. Future studies should focus on optimizing extraction and purification processes, evaluating the performance of arabinogalactan in complex formulations containing bioactive compounds, and assessing its scalability and economic feasibility for industrial applications.
